# Supplementary material for: Effect of a single one-hour teaching session about environmental pollutants and climate change on the understanding and behavioral choices of adolescents: The BREATHE pilot randomized controlled trial
Source: PLoS One. 2023 Nov 27;18(11):e0291199. doi: 10.1371/journal.pone.0291199 (PMC10681291; doi:10.1371/journal.pone.0291199)
Supplement: S1 File — (DOCX) [file pone.0291199.s001.docx]

**CLINICAL RESEARCH PROTOCOL**

**Behavioral Research of Environment and Air pollution Through Education (BREATHE) Study**

| Protocol Number: | 14-14683 |
| --- | --- |
| Principal Investigator: | Name: Mehrdad Arjomandi, MD  Telephone: (415) 221-4810 x 24393  E-mail: mehrdad.arjomandi@ucsf.edu |
| Co-Investigator | Name: Yorusaliem Abrham, MD  Telephone: (415) 221-4810 x 24269  E-mail: Yorusaliem.abrham@ucsf.edu |

**Study Sites:**

UCSF-Affiliated Site #1

Site name: San Francisco Veterans Affairs Medical Center

Address: 4150 Clement St, San Francisco, CA 94121

UCSF-Affiliated Site #2

Site name: University of California San Francisco Parnassus Campus

Address: 505 Parnassus Ave, San Francisco, CA 94143

Non-UCSF Community based Site #3

Site name: Piedmont Middle School

Address: 740 Magnolia Ave, Piedmont, CA 94611

Non-UCSF Community based Site #4

Site name: KIPP Heartwood Academy

Address: 1250 South King Road, San Jose, CA 95122

**TABLE OF CONTENTS**

[1 LIST OF ABBREVIATIONS 4](#_Toc149653500)

[2 PROTOCOL SYNOPSIS 5](#_Toc149653501)

[3 INTRODUCTION 7](#_Toc149653502)

[3.1 Preliminary Studies 7](#_Toc149653503)

[4 SIGNIFICANCE 8](#_Toc149653504)

[5 STUDY RATIONALE 8](#_Toc149653505)

[5.1 Risk/Benefit Assessment 9](#_Toc149653506)

[6 STUDY OBJECTIVES 9](#_Toc149653507)

[6.1 Primary Objective 9](#_Toc149653508)

[6.2 Secondary Objectives 9](#_Toc149653509)

[7 STUDY DESIGN 9](#_Toc149653510)

[7.1 Overview 9](#_Toc149653511)

[7.2 Questionnaire 9](#_Toc149653512)

[7.3 Teaching session 10](#_Toc149653513)

[8 CRITERIA FOR EVALUATION 10](#_Toc149653514)

[8.1 Efficacy Endpoints 10](#_Toc149653515)

[8.2 Safety Evaluations 10](#_Toc149653516)

[9 SUBJECT SELECTION 10](#_Toc149653517)

[9.1 Recruitment, Eligibility, and Study Population 10](#_Toc149653518)

[9.2 Vulnerable population 10](#_Toc149653519)

[9.3 Guardian/ parental consent 10](#_Toc149653520)

[9.4 Inclusion Criteria 11](#_Toc149653521)

[9.5 Exclusion criteria 11](#_Toc149653522)

[10 RANDOMIZATION 11](#_Toc149653523)

[11 BLINDING 11](#_Toc149653524)

[12 STUDY PROCEDURES AND GUIDELINES 11](#_Toc149653525)

[12.1 Clinical Assessments 11](#_Toc149653526)

[12.1.1 Demographics 11](#_Toc149653527)

[12.1.2 Clinical Laboratory Measurements 12](#_Toc149653528)

[12.2 Evaluations by visit 12](#_Toc149653529)

[13 STATISTICAL METHODS AND CONSIDERATIONS 13](#_Toc149653530)

[13.1 Sample size 13](#_Toc149653531)

[13.2 Measured variables. 13](#_Toc149653532)

[13.2.1 Independent variable 13](#_Toc149653533)

[13.2.2 Dependent Variables 13](#_Toc149653534)

[13.3 Covariates 13](#_Toc149653535)

[13.4 Statistical Models 13](#_Toc149653536)

[13.5 Interim Analysis 14](#_Toc149653537)

[13.6 Medical Monitoring 14](#_Toc149653538)

[14 TRIAL REGULATORY MATTERS 14](#_Toc149653539)

[15 ADVERSE EXPERIENCE REPORTING AND DOCUMENTATION 14](#_Toc149653540)

[15.1 Risk/Benefit Assessment 14](#_Toc149653541)

[16 QUESTIONNAIRE SCORING 15](#_Toc149653542)

# LIST OF ABBREVIATIONS

| **Behvr** | Behavior |
| --- | --- |
| **BREATHE** | Behavioral Research of Environment and Air Pollution Through Education |
| **HIPAA** | Health Insurance Portability and Accountability Act of 1996 |
| **ICF** | Informed consent form |
| **IRB** | Institutional Review Board |
| **KIPP** | Knowledge Is Power Program |
| **MID** | Minimally important difference |
| **RCT** | Randomized controlled trial |
| **SFVAMC** | San Francisco Veterans Affairs Health Care System |
| **UCSF** | University of California San Francisco |
| **Ustdg** | Understanding |

# PROTOCOL SYNOPSIS

| **Title** | Behavioral Research of Environment and Air pollution Through Education (BREATHE) Study |
| --- | --- |
| **Number of sites** | This is a single-center study involving two UCSF-affiliated sites and two community-based sites. |
| **Rationale** | Air pollution is a major environmental problem with important health consequences including cancer, cardiovascular, and pulmonary diseases that affects billions of people around the world. Multiple studies have shown air pollution in general and its individual components, such as ozone and particulate matter, to be associated with increased morbidity and mortality in humans. Despite the wealth of scientific literature on the health effects of air pollution, the public’s personal choices in their daily lives continues to be largely detrimental towards the environment, and the public support for generation of policies to regulate sources of pollution has remained modest at best. One potential cause of the disconnect between the state of scientific knowledge and the public and political support for aggressive regulation to protect the environment may be the lack of awareness and understanding of the available knowledge. However, there has been substantial media attention to the ongoing environmental problems in the recent years and, despite this publicity, the public and political support for the necessary steps to battle air pollution has remained low. An important impedance to the required support for environmental protection may be the established lifestyle demands of the current generations of adults. The current adult generations have mostly grown with little understanding of the human contribution to air pollution and their impact on the environment, making any substantial change to their behavior challenging.  A potential solution to this problem may be to focus on the future generations to see whether providing appropriate education may result in lifestyles that include beneficial behaviors towards the environment since their lifestyles and habits are as yet unformed and more amenable to influence. However, whether teaching the adolescents about the health effects of air pollution would affect their future understanding and awareness of air pollution or forms their future behavior to more environmentally sound life choices is unclear. |
| **Study design** | This is a randomized control clinical trial of efficacy of a one-hour teaching session on air pollution on the understanding and behavioral choices of adolescents. |
| **Primary objective** | The primary objective of this study is to determine whether a short interactive evidence-based teaching session on air pollution could shift the behavioral choices of adolescents towards environmentally friendlier options. |
| **Secondary objectives** | A secondary objective of the study is to determine short interactive evidence-based teaching session on air pollution improves understanding and awareness of air pollution in adolescents. |
| **Number of subjects** | 600 |
| **Subject selection criteria** | Inclusion Criteria:   1. 13-15 years of age. 2. Middle school student. 3. Ability to read, understand, and write in English (at the middle-school level). 4. Willingness to participate in follow-up visits of the study.   Exclusion criteria:   1. Learning disabilities such as autism. 2. Moving out of the area within the next 6 months. 3. Inability to complete pre-intervention questionnaire. |
| **Test product, dose, and route of administration** | One hour teaching session on air pollution, one time. |
| **Control product, dose, and route of administration** | One hour teaching session on vaccination, one time. |
| **Duration of subject participation and duration of study** | Completion of all study procedures will require an active time commitment of two and half **hours** over a period of twelve months. The breakdown of time commitment per In-Person Visit is mentioned below:   - Visit 1: 105 minutes   - Pre-teaching survey   - Teaching session including spirometry   - Post-teaching survey - Visit 2: 15 minutes. - Visit 3: 15 minutes. - Visit 4: 15 minutes.   Please note, participants completed assent and parental consent at home. This is not being included into the total active time commitment. |
| **Planned interim analyses** | The progress of the trial will be monitored as detailed in Data Safety and Monitoring Plan. An interim analysis will be performed to evaluate the reliability and validity of the study instrument, the questionnaire. |
| **Statistical analysis** | Data will be collected, entered into a database, and analyzed using Stata. The distribution of the data, and appropriate statistical measures will be calculated. The scores before and after the teaching sessions will be compared using paired-t-test. Multivariate regression analysis will be used to determine whether the independent variable is predictive of the outcome after adjustment for covariates. |
| **Rationale for Number of Subjects** | A sample size of 504 subjects (252 subjects in each group) will provide a power of 80% to detect a change in questionnaire score by t-test with a two-sided type I error of 0.05. Considering the drop-out rate, we proposed to recruit a total of 600 subjects (300 in each group). |

# INTRODUCTION

Air pollution continues to have proven negative impact on the health of children and adolescents. Air pollution is a major environmental problem with important health consequences including cancer and cardiopulmonary diseases that affects billions of people around the world. Multiple studies have shown air pollution in general and its individual components, such as ozone and particulate matter, to be associated with increased morbidity and mortality in humans. Despite the wealth of scientific literature on the health effects of air pollution, the public support for generation of policies and regulation of the sources of pollution has remained modest at best. One potential cause of this disconnect between the state of scientific knowledge and the public and political support for aggressive regulation for protection of environment may be the lack of awareness and understanding of the available knowledge. However, there has been substantial media attention to the ongoing environmental problems in the recent years, and despite this publicity, the public and political support for the necessary steps to battle air pollution remains low. Perhaps, an important impedance for the support is the established lifestyle demands of the current generation of adults. The current generations have grown with little understanding of environmental and air pollution, which may make substantial changes in their attitudes more difficult. Given above, an important way to affect the future personal choices and public support for necessary regulation and policies would be to provide adequate education to the children who will form the future generations of our society.

## Preliminary Studies

Multiple longitudinal studies have demonstrated the association between air pollution and reduced lung function (Lippmann et al., 2014, Dons et al., 2014, Fruin et al., 2014). A 2004 study from Gauderman and colleagues demonstrated the significant association between exposure to pollution in the form of NO2, acid vapor, elemental carbon, and particulate matter (PM2.5) and a reduction in forced expiratory volume in the first second (FEV1) (Gauderman et al., 2004). In particular, exposure to tropospheric ozone has been linked to an increase in bronchial hyperirritability in healthy individuals (as well as rhesus monkeys). Reducing exposure to pollution is of both a policy and public health importance. Focusing on public health, behavioral intervention is key for reducing individual exposure. Intervention has had success in positively impacting individual environmental behavior through various means, including an increase in curbside recycling (Schultz et al., 1999) and a reduction in the number of miles driven and trips taken during days of poor air quality (Henry et al., 2002).

# SIGNIFICANCE

Air pollution is a global public health issue affecting billions of people around the world. Air pollution is on the rise, and any intervention that could potentially improve air quality would be highly beneficial to the broader society. It is increasingly important to provide a method through which the public could be better educated about air pollution in a way that would positively impact their understanding and future behavior towards environmentally friendlier choices.

# STUDY RATIONALE

Air pollution is a major environmental problem with important health consequences including cancer, cardiovascular, and pulmonary diseases that affects billions of people around the world. Multiple studies have shown air pollution in general and its individual components, such as ozone and particulate matter, to be associated with increased morbidity and mortality in humans. Despite the wealth of scientific literature on the health effects of air pollution, the public’s personal choices in their daily lives continues to be largely detrimental towards the environment, and the public support for generation of policies to regulate sources of pollution has remained modest at best.

One potential cause of the disconnect between the state of scientific knowledge and the public and political support for aggressive regulation to protect the environment may be the lack of awareness and understanding of the available knowledge. However, there has been substantial media attention to the ongoing environmental problems in the recent years, and despite this publicity, the public and political support for the necessary steps to battle air pollution has remained low. An important impedance to the required support for environmental protection may be the established lifestyle demands of the current generations of adults. The current adult generations have mostly grown with little understanding of the human contribution to air pollution and their impact on the environment, making any substantial change to their behavior challenging.

A potential solution to this problem may be to focus on the future generations to see whether providing appropriate education may result in lifestyles that include beneficial behaviors towards the environment since their lifestyles and habits are as yet unformed and more amenable to influence. However, whether teaching the adolescents about the health effects of air pollution would affect their future understanding and awareness of air pollution or forms their future behavior to more environmentally sound life choices is unclear.

## Risk/Benefit Assessment

The risks of this study are minimal compared to potential benefits it holds for public health, both locally and globally. Air pollution is a significant and escalating problem affecting numerous countries. The findings of this study can inform the development of future interventions aimed at promoting environmental awareness and behavior change among adolescents and ultimately contribute to reduction of the global public health burden of air pollution.

# STUDY OBJECTIVES

## Primary Objective

The hypothesis of this study is that, educating adolescents about health effects of air pollution through a short interactive evidence-based teaching session will shape their behavior to make personal choices that are more environmentally friendly.

## Secondary Objectives

The secondary objective of the study is educating adolescents about health effects of air pollution through a short interactive evidence-based teaching session can improve their long-term understanding of human contribution to air pollution.

# STUDY DESIGN

## Overview

This is a double-blind randomized placebo-controlled clinical trial to examine the long-term efficacy of a short classroom-based teaching session about air pollution on the understanding and behavioral choices of middle school students, The study will be using a repeated measure design. A one-hour interactive script-based evidence-supported teaching curriculum about air pollution will be developed and its effects on climate change and human health.

Furthermore, to assess the effect of the teaching session, we developed a survey questionnaire with two topic domains to evaluate the understanding and future behavioral choices of the participating adolescents towards air pollution. We also developed a one-hour teaching session about health benefits of vaccination to be used as the control teaching session.

## Questionnaire

The questionnaire was developed to assess two domains: (1) adolescents’ understanding of air pollution, its sources, and its environmental and health effects and (2) adolescents’ future behavior based on their report of personal behavioral choices as well as their support for public policies with potentially important effects on air pollution, environment, and climate change (https://arjomandilab.ucsf.edu/questionnaires-powerpoints). It consisted of 15 questions which were divided into the two domains gauged towards quantification of adolescents’ understanding and behavioral choices.

The questionnaire is designed to generate two scores. The multiple-choice answers to each question were weighted to have a minimum score of 1 and a maximum score of 4 (Table 1). The understanding domain section contained 5 multiple-choice questions designed to target and quantify the understanding of the participants with a score ranging from a minimum of 5 to a maximum of 20 possible points. The behavior domain section contained 10 questions (7 multiple-choice and 3 free-narrative response) designed to target and quantify behavioral choices of participants with a score ranging from a minimum of 10 to a maximum of 38 possible points.

## Teaching session

The teaching sessions are one hour in duration, script-based, and associated with a PowerPoint slide presentation (https://arjomandilab.ucsf.edu/questionnaires-powerpoints). The intervention (air pollution) teaching session was developed by our research group based on the available scientific literature. The intervention session will also involve two activities:

- One will involve breathing through a straw to replicate the effect of breathing with lung inflammation.
- The second activity will be spirometry forcefully exhaling through an electronic spirometer to measure lung function.

# CRITERIA FOR EVALUATION

## Efficacy Endpoints

The study primary and secondary outcomes are listed below:

- The primary outcome of the study is the questionnaire behavioral domain score.
- The secondary outcomes are the questionnaire total and understanding domain score.

## Safety Evaluations

Not applicable.

# SUBJECT SELECTION

## Recruitment, Eligibility, and Study Population

Middle school students in the age range of 13 to 15 from two schools will be recruited from Piedmont Middle School and KIPP Heartwood Academy. Two to four weeks before the intervention is scheduled to take place, the participating teachers will be provided with a list of eligibility criteria, and will assess the eligibility of students, then provide the study personnel with a list of eligible students. These eligible students will be given the Information Letter and the Consent/Assent form to take home and complete. Those who complete the form and provide assent to participate in the study will be enrolled, and their eligibility will be further verified by study personnel through the demographic questionnaire.

## Vulnerable population

It is appropriate to include children in this study because the main purpose of this study is to focus on the efficacy of school-based intervention in changing the behavior of middle-school adolescents, and it is therefore essential to include children in this research so that the study population can properly reflect the target population.

## Guardian/ parental consent

The prospective student’s parents or guardians will receive information regarding their child’s invitation to participate in the study. They will be provided with a detailed description of the research study, including its nature and purpose, and asked to provide consent for their child’s participation. Only participants who have provided both assent and parental or guardian consent will be enrolled in the study, ensuring that participation is voluntary and contingent upon appropriate authorization.

## Inclusion Criteria

1. 13-15 years of age.
2. Middle school student.
3. Ability to read, understand, and write in English (at the middle-school level).
4. Willingness to participate in follow-up visits of the study.

## Exclusion criteria

1. Learning disabilities (such as autism).
2. Moving out of the area within the next 6 months
3. Inability to complete pre-intervention questionnaire.

# RANDOMIZATION

In this randomized control trial, eligible students will be randomized by block randomization, as explained below. The students will be assigned a study ID number through sorting their last names (and first names if necessary) alphabetically and then assigned a study ID number. They will then be partitioned by simple randomization of their subject ID number within their classrooms using STATA software to either receive a one-hour script-based teaching on either the effects of air pollution on lung health (intervention group) or the role of vaccination in public health (control group), such that about equal numbers of students in each classroom were assigned to intervention and control groups.

# BLINDING

In this study, blinding of participants and study staff to the intervention will not be feasible. However, to minimize bias, the data from the questionnaires will be entered into a database by two research assistants who will also be blinded to the assignment of the participants. Furthermore, the scoring of the free-narrative survey responses will be carried out by two observers who will be blinded to the participant assignment. These observers will adhere to the pre-defined guidelines that categorizes response from no response or an irrelevant response to a relevant response or a response related to a recent relevant event. This blinding procedure will ensure that the scoring of free-narrative responses and the data entry into the databases are conducted objectively and without knowledge of the participant assignment.

# STUDY PROCEDURES AND GUIDELINES

Prior to conducting any study-related activities, written informed consent and the Health Insurance Portability and Accountability Act (HIPAA) authorization will be signed by the parent or guardian and we will also get an assent from the participant.

## Clinical Assessments

### Demographics

Demographic information (date of birth, gender, race, family income) will be recorded at Visit 1.

### Clinical Laboratory Measurements

Not applicable

## Evaluations by visit

Participants will be asked to complete a total of three in-person visits and one at-home over a period of at least 12 months.

Acquisition of Informed Consent: Parents/guardians of students who meet the eligibility criteria will be sent a set of papers containing:

- A letter explaining the purpose and methods of the study, and
- A letter of consent and assent for both the parent and student to sign.

All documents will be written in simple English and Spanish (<8th grade level). These forms will be brought to school, and those students with completed consent/assent forms will be eligible to participate in the study.

To ensure understanding of the consent and assent material, study staff will review the consent document over the phone with the potential subjects/ guardian.

**Visit 1**

- Questionnaire: The enrolled students will complete a survey (15 multiple-choice questions; five targeting understanding (score range 5-20); ten targeting behavioral choices (score range 10-38)) designed to evaluate their understanding and predict their future behavior towards air pollution immediately before the intervention.
- Teaching session: participants will be given a one-hour script-based teaching on either the effects of air pollution on lung health (intervention group) or the role of vaccination in public health (control group). The teaching session also includes offering students to breathe through a straw and also undergo spirometry.
- Questionnaire: The enrolled students will complete the same survey immediately after the intervention.

**Visit 2**

- Questionnaire: The enrolled students will complete the same survey in about three months after the intervention.

**Visit 3**

- Questionnaire: The enrolled students will complete the same survey in about six months after the intervention

**Visit 4**

- Questionnaire: The enrolled students will complete the same survey in about twelve months after the intervention

Due to scholastic schedules and holidays the questionnaires may be applied within 8 weeks of the planned schedule (for example 1 to 5 months or 4 to 8 months or 10 to 14 months).

# STATISTICAL METHODS AND CONSIDERATIONS

Data will be collected, entered into a database, and analyzed using statistical software. The distribution of the data, and the appropriate statistical measures will be calculated. The questionnaire scores before and after the teaching sessions will be compared using paired-t-test. In addition, multivariable regression analysis will be performed to determine whether or not the independent variable is predictive of the outcome and to determine the contribution of covariates.

## Sample size

For sample size and power calculation, we made the following assumptions:

- Minimally important difference in questionnaire score of 10% change due to the intervention.
- Standard deviation of change in score of 40%.
- A drop-out rate of about 20% due to factoring including movement of subjects out of the area.

A sample size of 504 subjects (252 subjects in each group) will provide a power of 80% to detect a change in questionnaire score by t-test with a two-sided type I error of 0.05. Considering the drop-out rate, we proposed to recruit a total of 600 subjects (300 in each group).

## Measured variables.

### Independent variable

- One hour teaching session about air pollution and climate change

### Dependent Variables

- Behavior domain score (primary outcome).
- Total domain score (secondary outcome).
- Understanding domain score (secondary outcome)

## Covariates

- Age
- Sex
- Income level
- Race
- Ethnicity
- Assigned classrooms.

## Statistical Models

- Paired t-test comparison of pre versus post teaching session.
- Linear regression modeling analysis will be performed to compare the scores and change in them between the intervention and control group with adjustment for covariates including age, sex, race, ethnicity, income level, and assigned classrooms as appropriate.
- Multivariate generalized estimating equation regression analysis will be performed to determine whether there is an incremental effect from “time” on scores and change in scores with adjustment for covariates including age, sex, race, ethnicity, income level, and assigned classrooms as appropriate.

## Interim Analysis

The progress of the trial will be monitored as detailed in Data Safety and Monitoring Plan. An interim analysis to evaluate the reliability and validity of the study instrument, the survey questionnaire will be performed.

## Medical Monitoring

Internally, the investigator and co-investigator will perform the quarterly safety reviews. Externally, a Data Safety and Monitoring Board (DSMB) has been created to evaluate the study with respect to the following:

- Participant safety, burden, confidentiality, and any other matter pertaining to protection of study subjects.
- Quality control, including follow-up for events, and Study productivity in terms of significant research results in addressing the primary study aims.

The DSMB members will include two senior investigators not directly involved with the study.

# TRIAL REGULATORY MATTERS

The University of California San Francisco (UCSF) Institutional Review Board (IRB) and the San Francisco Veterans Affairs Health Care System (SFVAHCS) Committee on Research and Development approved the study protocols. The study is registered with the United States (U.S.) National Library of Medicine (Behavioral Research of Environment and Air Pollution Through Education (BREATHE) study; ClinicalTrials.gov identifier NCT02471872).

# ADVERSE EXPERIENCE REPORTING AND DOCUMENTATION

This study is a category 7 low risk behavioral research, and it will be performed under no greater than minimum risk (45 CFR 46.404,21 CFR 50.51).

## Risk/Benefit Assessment

The risks of this study are minimal compared to potential benefits it holds for public health, both locally and globally. Air pollution is a significant and escalating problem affecting numerous countries. The findings of this study can inform the development of future interventions aimed at promoting environmental awareness and behavior change among adolescents and ultimately contribute to reduction of the global public health burden of air pollution.

# QUESTIONNAIRE SCORING
