# Supplementary material for: Effect of a single one-hour teaching session about environmental pollutants and climate change on the understanding and behavioral choices of adolescents: The BREATHE pilot randomized controlled trial
Source: PLoS One. 2023 Nov 27;18(11):e0291199. doi: 10.1371/journal.pone.0291199 (PMC10681291; doi:10.1371/journal.pone.0291199)
Supplement: S2 File — S1 Table presented the factor loadings from factor analysis with two factors for the 15 questions of the questionnaire. S2 Table presented the factor loadings from factor analysis with five factors for the 15 questions of the questionnaire. S3 Table presented the results from multivariable regression analyses using generalized estimating equations with repeated measure design. (DOCX) [file pone.0291199.s002.docx]

**SUPPLEMENTAL TABLES**

Effect of a single one-hour teaching session about environmental pollutants and climate change on the understanding and behavioral choices of adolescents: the BREATHE pilot randomized controlled trial

Yorusaliem Abrham ^1,2^ , Siyang Zeng ^1,2,3^ , Rachel Tenney^1,2,4^, Caroline Davidson ^1,5^, Emily Yao ^1,2^, Chantal Kloth ^1,2^, Sarah Dalton ^1,2^, Mehrdad Arjomandi ^1,2⁎^

^1^ San Francisco Veterans Affairs Health Care System, San Francisco, California, USA

^2^ Department of Medicine, University of California, San Francisco, California, USA

^3^ Department of Biomedical Informatics and Medical Education, University of Washington, Seattle

^4^ Department of Medicine, Weil Cornell University Medical Center, New York, New York, USA

^5^ National Council for Mental Wellbeing, New York, New York, USA

Table of Contents

[Supplemental Table S1 3](#_Toc143870731)

[Supplemental Table S2 4](#_Toc143870732)

[Supplemental Table S3 5](#_Toc143870733)

# Supplemental Table S1

**Factor-matrix of two factors for the 15 questions of the questionnaire.**

| **Question** | **Domain** | **Factor 1** | **Factor 2** |
| --- | --- | --- | --- |
| 6 | B | 0.790 | 0.176 |
| 10 | B | 0.519 |  |
| 14 | B | 0.464 |  |
| 7 | B | 0.391 |  |
| 8 | B | 0.317 |  |
| 13 | B | 0.300 |  |
| 9 | B | 0.280 | -0.137 |
| 1 | U |  | 0.659 |
| 4 | U |  | 0.526 |
| 3 | U |  | 0.369 |
| 5 | U | 0.202 | 0.285 |
| 2 | U |  | 0.251 |
| 15 | B | 0.116 | 0.356 |
| 11 | B |  | 0.298 |
| 12 | B |  | 0.172 |
|  |  |  |  |
| Cumulative % of variance explained | | 10.7 | 19.4 |

Footnote: Factor loadings from factor analysis with “varimax” rotation for two factors were presented. Future behavior and understanding domains were represented by “B” and “U”, respectively. Empty cells represent zero loadings.

# Supplemental Table S2

**Factor-matrix of five factors for the 15 questions of the questionnaire.**

| **Question** | **Domain** | **Factor 1** | **Factor 2** | **Factor 3** | **Factor 4** | **Factor 5** |
| --- | --- | --- | --- | --- | --- | --- |
| 6 | B | 0.955 | 0.157 | 0.144 | 0.122 | -0.151 |
| 10 | B | 0.460 |  |  |  | 0.151 |
| 7 | B | 0.311 |  |  |  | 0.180 |
| 8 | B | 0.281 |  |  |  |  |
| 1 | U | -1.161 | 0.942 |  | 0.222 |  |
| 4 | U |  | 0.409 |  |  | -0.123 |
| 3 | U | 0.150 | 0.342 | -0.159 |  |  |
| 5 | U | 0.202 | 0.287 | 0.133 | -0.128 | 0.151 |
| 15 | B | 0.190 | 0.293 | -0.242 |  |  |
| 11 | B |  | 0.236 | -0.162 |  | 0.194 |
| 9 | B | 0.117 |  | 0.981 | 0.136 |  |
| 2 | U |  |  | -0.339 | 0.150 |  |
| 13 | B | 0.215 | -0.125 |  | 0.962 |  |
| 14 | B | 0.437 |  |  |  | 0.888 |
| 12 | B |  | 0.157 |  |  | 0.129 |
|  |  |  |  |  |  |  |
| Cumulative % of variance explained | | 11.2 | 21.0 | 29.4 | 36.6 | 43.1 |

Footnote: Factor loadings from factor analysis with “varimax” rotation for five factors were presented. Future behavior and understanding domains were represented by “B” and “U”, respectively. Empty cells represent zero loadings.

# Supplemental Table S3

**Results from multivariable regression analyses using generalized estimating equations with repeated measure design.**

| **Group** | **Independent variables** | **Parameter estimate** | **95% confidence interval** | | **P value** |
| --- | --- | --- | --- | --- | --- |
|  |  |  | Lower bound | Upper bound |  |
| **Dependent variable: Total score** | | | | | |
| All participants | (Intercept) | 43.34 | 9.05 | 77.63 | 0.013 |
|  | Immediate post | 1.35 | 0.40 | 2.29 | 0.005 |
|  | 1-month post | 2.40 | 1.22 | 3.57 | <0.001 |
|  | Age | -0.56 | -3.15 | 2.02 | 0.669 |
|  | Sex | -0.27 | -2.59 | 2.05 | 0.822 |
|  | Hispanic | 5.42 | 3.15 | 7.69 | <0.001 |
| Intervention | (Intercept) | 54.29 | 10.59 | 97.99 | 0.014 |
|  | Immediate post | 2.20 | 0.93 | 3.47 | <0.001 |
|  | 1-month post | 2.28 | 0.89 | 3.67 | 0.001 |
|  | Age | -1.50 | -4.81 | 1.80 | 0.372 |
|  | Sex | 1.81 | -1.54 | 5.16 | 0.289 |
|  | Hispanic | 7.20 | 4.97 | 9.43 | <0.001 |
| Control | (Intercept) | 30.43 | -9.94 | 70.81 | 0.139 |
|  | Immediate post | 0.57 | -0.75 | 1.89 | 0.397 |
|  | 1-month post | 2.52 | 0.64 | 4.41 | 0.008 |
|  | Age | 0.48 | -2.54 | 3.51 | 0.754 |
|  | Sex | -1.79 | -4.76 | 1.18 | 0.236 |
|  | Hispanic | 2.69 | -0.44 | 5.82 | 0.091 |
| **Dependent variable: Understanding score** | | | | | |
| All participants | (Intercept) | 19.65 | -0.19 | 39.48 | 0.052 |
|  | Immediate post | 0.24 | -0.34 | 0.82 | 0.414 |
|  | 1-month post | -0.141 | -0.88 | 0.60 | 0.710 |
|  | Age | -0.26 | -1.76 | 1.24 | 0.735 |
|  | Sex | -0.17 | -1.35 | 1.01 | 0.776 |
|  | Hispanic | 2.19 | 1.21 | 3.17 | <0.001 |
| Intervention | (Intercept) | 26.30 | -2.10 | 54.71 | 0.069 |
|  | Immediate post | 0.34 | -0.45 | 1.13 | 0.396 |
|  | 1-month post | -0.50 | -1.52 | 0.53 | 0.343 |
|  | Age | -0.79 | -2.97 | 1.39 | 0.476 |
|  | Sex | 0.20 | -1.84 | 2.24 | 0.848 |
|  | Hispanic | 2.66 | 1.16 | 4.16 | <0.001 |
| Control | (Intercept) | 11.43 | -7.27 | 30.13 | 0.230 |
|  | Immediate post | 0.16 | -0.70 | 1.01 | 0.717 |
|  | 1-month post | 0.19 | -0.86 | 1.25 | 0.718 |
|  | Age | 0.37 | -0.98 | 1.73 | 0.588 |
|  | Sex | -0.25 | -1.64 | 1.13 | 0.718 |
|  | Hispanic | 1.58 | 0.37 | 2.80 | 0.010 |
| **Dependent variable: Behavior score** | | | | | |
| All | (Intercept) | 23.69 | 3.51 | 43.87 | 0.021 |
|  | Immediate post | 1.10 | 0.34 | 1.87 | 0.004 |
|  | 1-month post | 2.54 | 1.57 | 3.51 | <0.001 |
|  | Age | -0.30 | -1.81 | 1.20 | 0.691 |
|  | Sex | -0.10 | -1.79 | 1.60 | 0.912 |
|  | Hispanic | 3.23 | 1.43 | 5.03 | <0.001 |
| Intervention | (Intercept) | 27.99 | 6.09 | 49.89 | 0.012 |
|  | Immediate post | 1.86 | 0.73 | 2.98 | 0.001 |
|  | 1-month post | 2.78 | 1.69 | 3.87 | <0.001 |
|  | Age | -0.71 | -2.31 | 0.88 | 0.380 |
|  | Sex | 1.61 | -0.44 | 3.66 | 0.124 |
|  | Hispanic | 4.54 | 3.06 | 6.03 | <0.001 |
| Control | (Intercept) | 19.00 | -12.84 | 50.84 | 0.242 |
|  | Immediate post | 0.41 | -0.54 | 1.36 | 0.395 |
|  | 1-month post | 2.33 | 0.75 | 3.91 | 0.003 |
|  | Age | 0.11 | -2.28 | 2.50 | 0.929 |
|  | Sex | -1.54 | -3.92 | 0.84 | 0.205 |
|  | Hispanic | 1.11 | -1.30 | 3.51 | 0.365 |
| **Dependent variable: Change in total score** | | | | | |
| All participants | (Intercept) | -1.84 | -22.13 | 18.45 | 0.858 |
|  | Intervention | 1.08 | -0.68 | 2.83 | 0.230 |
|  | Age | 0.30 | -1.17 | 1.76 | 0.693 |
|  | Sex | -1.44 | -3.11 | 0.24 | 0.092 |
|  | Hispanic | -1.59 | -3.49 | 0.30 | 0.099 |
| **Dependent variable: Change in understanding score** | | | | | |
| All participants | (Intercept) | 6.24 | -9.28 | 21.76 | 0.430 |
|  | Intervention | -0.26 | -1.49 | 0.98 | 0.684 |
|  | Age | -0.42 | -1.56 | 0.72 | 0.467 |
|  | Sex | -0.60 | -1.75 | 0.56 | 0.313 |
|  | Hispanic | -0.22 | -1.45 | 1.00 | 0.721 |
| **Dependent variable: Change in behavior score** | | | | | |
| All participants | (Intercept) | -8.08 | -25.68 | 9.53 | 0.368 |
|  | Intervention | 1.33 | -0.07 | 2.74 | 0.063 |
|  | Age | 0.72 | -0.57 | 2.01 | 0.275 |
|  | Sex | -0.84 | -2.19 | 0.51 | 0.221 |
|  | Hispanic | -1.37 | -3.37 | 0.63 | 0.179 |

Footnote: From each multivariable regression models using generalized estimating equations with repeated measure design, the dependent variable, data from the group of participants, and the independent variables with their corresponding results (parameter estimate, lower and upper bound of the 95% confidence interval, and P value) were presented.
