# Supplementary material for: Effect of a single one-hour teaching session about environmental pollutants and climate change on the understanding and behavioral choices of adolescents: The BREATHE pilot randomized controlled trial
Source: PLoS One. 2023 Nov 27;18(11):e0291199. doi: 10.1371/journal.pone.0291199 (PMC10681291; doi:10.1371/journal.pone.0291199)
Supplement: Attachment — Submitted filename: Breathe_pbP_PLOS_081023_v7.1.docx [file pone.0291199.s003.docx]

Dear Dr. Wade,

Thank you for considering our manuscript “Effect of a single one-hour teaching session about environmental pollutants and climate change on the understanding and behavioral choices of adolescents: the BREATHE randomized controlled trial” for publication in PLOS ONE. We have revised the manuscript according to the reviewers’ comments and are providing a point-by-point response below. We hope that the editors and reviewers will find the revised manuscript suitable for publication.

Thank you and best regards,

Mehrdad Arjomandi on behalf of co-authors

**GENERAL COMMENTS**

*1- COMMENT: We recommend that you deposit your laboratory protocols in protocols.io to enhance the reproducibility of your results. Protocols.io assigns your protocol its own identifier (DOI) so that it can be cited independently in the future.*

RESPONSE: We have deposited the protocol for BREATHE study on Protocols.io server and is awaiting the assignment of a DOI.

*2- COMMENT: You have indicated that data from this study are available upon request. PLOS only allows data to be available upon request if there are legal or ethical restrictions on sharing data publicly.*

RESPONSE: We have uploaded the study data to Dryad and have provided a digital object identifier (DOI): doi:10.7272/Q63T9FGB.

*3- COMMENT: We note that you have included the phrase “data not shown” in your manuscript. Unfortunately, this does not meet our data sharing requirements.*

RESPONSE: We have uploaded all the study data to Dryad and have provided a digital object identifier (DOI): doi:10.7272/Q63T9FGB.

**COMMENTS TO THE AUTHOR**

**REVIEWER#1**

*1- COMMENT: Detailed inclusion and exclusion criteria [are to be provided].*

RESPONSE: We have revised the Methods section of the manuscript to clearly outline the inclusion/exclusion criteria, as described below:

Methods section, Study population- Page 10-11

“**Study Population**

The inclusion criteria for participation in the study included (1) being a middle school students aged 13 to 15 years of age; (2) ability to read, understand, and write in English at the middle school level; and (3) willingness to participate in the follow-up visits of the study. The exclusion criteria included (1) inability to complete the pre-intervention questionnaire; and (2) known plans to move out of the area within the next 6 months after enrollment in the study. As an initial attempt, teachers from the San Jose, California metropolitan area KIPP school (Knowledge Is Power Program), a national network of free open-enrollment college preparatory public schools dedicated towards students in underserved communities, were invited to participate in the study by collaborating with study recruitment and educational activities.

…

Beginning February 1, 2015, students from all three 8th grade classrooms at the San Jose KIPP school were invited by their teachers to participate in the study. The teachers then distributed a letter of invitation along with assent and consent forms to the interested students to take home and review with their parents/guardians, and later collected and provided the forms to the study investigators. Students who provided signed consent and assent forms were included in the study if they met the enrollment criteria. The consent and ascent forms were provided in English and Spanish language to allow for language preference of the students’ parents and/or guardians in this school with majority Hispanic population. However, the teaching sessions and the questionnaire administered to the students were only in English, as the KIPP students were considered to all be proficient in reading and writing English language at the level of middle school. The study enrollment ended on December 20, 2018.”

*2- COMMENT: Detailed information on the questionnaires such as validation, reliability, the interpretation of the scores, and the sample size calculation are to be provided.*

RESPONSE: We have provided further text in the Methods and Results sections to describe the reliability and validation testing methodology and results, sample size and power calculation, and the scoring and interpretation of the questionnaire responses, as described below:

Methods section, Questionnaire- Page 14-16

“**Questionnaire**

The questions on the questionnaire were formulated collaboratively by our research team that included researchers and educators during several discussion sessions. The questionnaire was specifically developed for this study on the basis of the hypothesis that providing knowledge that result in improved risk perception and outcome expectancy principles towards environmental health, as outlined in Social Cognitive Theory, is best achieved in adolescent age group. The questions were carefully crafted, considering the age group of the target population (middle adolescents in 13 to 15 years of age) and the current environmental issues, and were designed to provide a quantification of perspective participants’ level of understanding environmental health issues as well as degree of interest in making lifestyle choices that may be perceived as environmentally friendly. To do this, the questions were designed to assess several components, including knowledge of environmental pollution and its health effects, perception of how much participants think and discuss environmental issues, and attitudes towards daily activities and lifestyle choices aimed at mitigating air pollution and climate change.

The questionnaire was developed to assess two domains: (1) adolescents’ understanding of air pollution, its sources, and its environmental and health effects and (2) adolescents’ future behavior based on their report of personal behavioral choices as well as their support for public policies with potentially important effects on air pollution, environment, and climate change (https://arjomandilab.ucsf.edu/questionnaires-powerpoints). It consisted of 15 questions which were divided into the two domains gauged towards quantification of adolescents’ understanding and behavioral choices. The questionnaire generated two scores, which were weighted sums of the questions in their perspective sections as described below. The multiple-choice answers to each question were weighted to have a minimum score of 1 and a maximum score of 4. …

…

The structural validity (reliability) of the questionnaire was evaluated by computing the correlation coefficient of the data collected before and immediately after the teaching sessions from all participants. To account for any potential effects from the intervention administered, the correlation coefficient of the data was also computed within the control group. The content validity was evaluated by factor analysis with “varimax” rotation using data collected from all participants before the teaching sessions. Barlett’s test of sphericity was performed to test whether data was appropriate for factor analysis. The number of factors to extract from factor analysis was determined using Scree plot and parallel analysis. Factor loadings and percentage of variance explained by the factors were reported.”

Methods section, Sample Size and Power Calculations- Page 16

**“Sample Size and Power Calculation**

For sample size and power calculation, we made the following assumptions: (1) minimally important difference in questionnaire score of 10% change due to the intervention, (2) standard deviation of change in score of 40%, and (3) a drop-out rate of about 20% due to factoring including movement of subjects out of the area. A sample size of 504 subjects (252 subjects in each group) will provide a power of 80% to detect a change in questionnaire score by t-test with a two-sided type I error of 0.05. Considering the drop-out rate, we proposed to recruit a total of 600 subjects (300 in each group).”

Results section - Page 23-24

**“Questionnaire Performance**

…

The test-retest reliability of the questionnaire computed using data collected from all participants before and immediately after the teaching sessions was 0.77. The test-retest reliability was 0.75 when it was computed using data from the control group only. Barlett’s test of sphericity showed a significant P value <0.001and thus granted appropriateness of factor analysis. Scree plot and parallel analysis of data suggested factor analysis with two factors. This analysis showed that using two factors separated the survey questions in accordance with the understanding and future behavior domains but explained only 19.4% of the variance (**Supplemental Table S1**). We subsequently performed factor analyses for three, four, and five factors. With five factors, the percentage of variance explained by the factors improved to be 43.1% with survey questions quantifying behaviors loaded mostly on Factors 1, 3 and 4; while questions quantifying understanding loaded mainly on Factor 2 (**Supplemental Table S2**).”

*3- COMMENT: Based on the CONSORT statement, all statistical tests for baseline comparison should be avoided.*

RESPONSE: We have removed the statistical tests of baseline measures from **Table 1** of the manuscript.

*4- COMMENT: Page 21 Questionnaire performance and based on Figure 2, the median/IQR scores is to be provided.*

RESPONSE: We have provided the appropriate descriptive statistics including medians, IQR, and total range in **Table 2**.

*5- COMMENT: Detailed regression analysis output to be presented in table form.*

RESPONSE: We have provided detailed description of the regression modeling analysis and an additional table describing its findings in **Table 5**.

*6- COMMENT: Page 18, the statement P=0.036 by regression analysis with adjustment for age, sex, and ethnicity)’ is to be separated and presented in another section of the results.*

RESPONSE: We have revised the manuscript accordingly.

Results section- Page 26-27

“The changes in questionnaire scores between the intervention and the control groups were analyzed using multivariable linear regression analyses. These analyses showed that the changes in behavior scores were statistically significantly higher in the intervention group compared to the control group (a differentially larger change [95% CI] of 1.7 [0.1, 3.3] in the intervention group; P=0.037) at immediately post-teaching (**Fig. 3** and **Table 5**).”

*7- COMMENT: Table 3, 95% CI is to be included.*

RESPONSE: We have provided the data as requested- now **Table 4**.

*8- COMMENT: The statement ‘the limitation of our sample size’ is to be justified with sample size calculation.*

RESPONSE: We have provided a description of the sample size and power calculation as requested- as described above in response to another comment. In addition, we have clarified our point about the limitation of the sample size in the Discussion section, as described below.

Limitations section- Page 34

“… Finally, there was a limited diversity of the income level among the population studied with about two thirds of the participants reporting annual income levels below $50,000 (at or near poverty level in the US). This limitation may adversely affect the generalizability of the study findings, as the income level could potentially influence people’s behavior towards environment. However, our study was not powered to examine the contribution of socioeconomic factors in efficacy of the intervention administered. Future research could explore the role of socioeconomic factors in the effectiveness of educational interventions promoting pro-environmental behavior in adolescents.”

*9- COMMENT: In the study protocol, a number of other independent variables and potential confounding variables/covariates, and different statistical approach was mentioned. The differences are to be indicated and whether those variables were considered in this study and the analysis.*

RESPONSE: We analyzed the data using both t-test comparison and regression modeling (GEE) with adjustment for covariates. These two methods of analysis provided similar findings albeit with slightly different statistical significance level. Because t-test comparisons are the more common approach being used in the analysis of data from clinical trials and simpler to understand for the audience, we chose to present the t-test comparisons as the primary methods of analysis. We now have described the results of regression analysis in the manuscript and included its results in the Supplemental Appendix as well.

Methods section- Page 17-18

“The distribution of total scores, as well as scores from understanding and behavior domains of the questionnaire, were examined both within-participants and between intervention and control groups. The differences in the scores from before to immediate or 1-month after the intervention within each participant were examined using paired t-test. Changes in scores within each participant from before to immediate or 1-month after the intervention were obtained by subtracting the baseline scores from the later scores, respectively. The changes in questionnaire scores (outcomes) within each group were examined using t-test comparisons. Regression analyses using generalized estimating equations (GEE) were also performed to examine the changes in outcomes with a repeated measure design and adjustment for covariates including age, sex, race, ethnicity, income level, and assigned classrooms as appropriate. Given that only few students provided race classification (22 out of 77) and the skewed distribution of family income levels of participants, only age, sex, and ethnicity were included in the final regression modeling. Furthermore, linear regression modeling with adjustment for covariates was performed for comparison of changes between the intervention and the control group at different time points.”

Results section- Page 25-27

"Similar results were generated when the changes in questionnaire scores within each group were analyzed using paired t-test or multivariable regression (GEE) (GEE results presented in **Supplemental Table S3**). The teaching sessions did not result in any significant change in the participants’ understanding domain scores in either the intervention or the control groups (**Fig. 2**, red box plots and **Table 4**). However, the intervention (air pollution) teaching session did result in a significant and important increase in behavior domain score from baseline to immediately post-teaching, which continued to be present at 1-month follow up (**Fig. 2**, panel A, blue box plots and **Table 4**). The control (vaccine) teaching session did not cause any significant change in the behavior domain score immediately post-teaching (**Fig. 2**, panel B, blue box plots and **Table 4**).

The changes in questionnaire scores between the intervention and the control groups were analyzed using multivariable linear regression analyses. These analyses showed that the changes in behavior scores were statistically significantly higher in the intervention group compared to the control group (a differentially larger change [95% CI] of 1.7 [0.1, 3.3] in the intervention group; P=0.037) at immediately post-teaching (**Fig. 3** and **Table 5**).”

*10- COMMENT: The citation in the text did not comply with the journal format.*

RESPONSE: We have revised the manuscript to include the journal formatting requirements.

**REVIEWER #2**

*1- COMMENT: While an important activity I do not think that the study design described by the authors is the one the test out their hypotheses.*

*I am a bit confused by the control group. I one hour teaching session was given to each group of different topics. I am not sure if this shows that the 1-hour teaching is effective of it has to do with the subject matter. I would think that the control group would be reading material of about air pollution and compare that to the teaching session.*

*I am unclear as to why the authors chose 2 different subject to perform an interactive teaching session on. This is more testing if the study population cares more about vaccinations or air pollution not whether the interactive teaching method is effective. If the authors wanted to test that (as their title suggests) than the control group should either receive the information in a different way, such as only reading material, or none at all. Additionally, 8th graders may not have a real choice on whether they get vaccinated or not so this is a bit of an odd choice.*

RESPONSE: We acknowledge the reviewer’s concern regarding the choice of the control group. The main research question was whether educating adolescent about air pollution would result in behavioral changes that are environmentally friendly. To address this question, we chose an in-person teaching session about air pollution by researchers. The hypothesis was not to test the researchers’ presence in a teaching session, but rather what the researchers taught. Thus, an in-person teaching session by researchers about another topic other than air pollution (in this case vaccination) is indeed an appropriate control strategy. Giving reading materials about air pollution would have been an appropriate control strategy to test the hypothesis that the presence of the researchers and in-person nature of the teaching will affect the adolescents’ behavior, but not the role of teaching about air pollution on their behavior.

We revised the methods section in the manuscript and have added the following paragraph to clarify this matter.

Methods section, Page 13

“Because the main objective of this study was to determine whether educating adolescents about air pollution in a form of a single teaching session would result in behavioral changes that are environmentally friendly, we chose a one-hour in-person teaching session about air pollution by researchers as the intervention. For control group, we chose to provide an in-person teaching session by researchers about another topic other than air pollution (vaccination). This allowed us to test the hypothesis that what the researchers taught (versus the researchers’ presence) will affect the adolescents’ future behavior.”

*2- COMMENT: There are also a lot of missing details on how the authors developed the questionnaire and how they designed their teaching sessions.*

*Where did the questions for the questionnaire come from? It says from the research group but is there existing literature that shows that these questions are getting the information that the authors want. In other words, were this questionnaire previously validated?*

RESPONSE: We have revised the manuscript to include further details about the development and scoring of the questionnaire.

Methods section, Page 14-16:

**“Questionnaire**

The questions on the questionnaire were formulated collaboratively by our research team that included researchers and educators during several discussion sessions. The questionnaire was specifically developed for this study on the basis of the hypothesis that providing knowledge that result in improved risk perception and outcome expectancy principles towards environmental health, as outlined in Social Cognitive Theory, is best achieved in adolescent age group. The questions were carefully crafted, considering the age group of the target population (middle adolescents in 13 to 15 years of age) and the current environmental issues, and were designed to provide a quantification of perspective participants’ level of understanding environmental health issues as well as degree of interest in making lifestyle choices that may be perceived as environmentally friendly. To do this, the questions were designed to assess several components, including knowledge of environmental pollution and its health effects, perception of how much participants think and discuss environmental issues, and attitudes towards daily activities and lifestyle choices aimed at mitigating air pollution and climate change.

The questionnaire was developed to assess two domains: (1) adolescents’ understanding of air pollution, its sources, and its environmental and health effects and (2) adolescents’ future behavior based on their report of personal behavioral choices as well as their support for public policies with potentially important effects on air pollution, environment, and climate change (https://arjomandilab.ucsf.edu/questionnaires-powerpoints). It consisted of 15 questions which were divided into the two domains gauged towards quantification of adolescents’ understanding and behavioral choices. The questionnaire generated two scores, which were weighted sums of the questions in their perspective sections as described below. The multiple-choice answers to each question were weighted to have a minimum score of 1 and a maximum score of 4…

…

The structural validity (reliability) of the questionnaire was evaluated by computing the correlation coefficient of the data collected before and immediately after the teaching sessions from all participants. To account for any potential effects from the intervention administered, the correlation coefficient of the data was also computed within the control group. The content validity was evaluated by factor analysis with “varimax” rotation using data collected from all participants before the teaching sessions. Barlett’s test of sphericity was performed to test whether data was appropriate for factor analysis. The number of factors to extract from factor analysis was determined using Scree plot and parallel analysis. Factor loadings and percentage of variance explained by the factors were reported.”

*3- COMMENT: The authors seem to be conflating two different but related issues: support for air pollution reduction policies/regulations and behavior changes. Given the premise of the study I recommend focusing on the behavioral changes and possible mention how that can lead to increased awareness and policy changes in the future in the discussion.*

RESPONSE: We agree with the reviewer and have revised the manuscript accordingly.

Introduction section, Page 8

" One potential factor associated with the disconnect between the state of the scientific knowledge and the public’s behavioral and lifestyle choices towards the environment may be due to a lack of awareness and understanding of the available knowledge. While there has been substantial media attention and increased publicity to the ongoing environmental problems in the recent years, the level of public enthusiasm and motivation to take the necessary steps to battle air pollution by modifying their behavior and lifestyle has remained low… ”

Conclusion section, Page 34

“… The findings of this study can inform the development of future interventions aimed at promoting environmental awareness and behavior change among adolescents and ultimately contribute to public and political support for aggressive regulation to protect the environment that could then result in reduction of the global public health burden of air pollution.”

*4- COMMENT: There should be some background on how interactive teaching can be effective. There needs to be some citation on environmental education. For example, why did the authors choose interactive teaching session of 1 hour? Has education shown to be effective in changes behaviors?*

RESPONSE: We have revised the Discussion section to address the above concern.

Discussion section, Page 30

“A few other studies have reported examining the effect of environmental education in form of an intervention and examining its effects on youth; however, these studies were usually of a much longer duration teaching, were administered to older youth in the high school through college age range, were not evaluating specific environmentally relevant behavioral changes, and/or lacked appropriate control arm. For example, a 2019 study of 578 secondary school students in the 15 to 16 years of age range in Southeast Asia (Laos or Lao People's Democratic Republic) has documented that environmental education over a semester (3 sessions over 2 weeks as part of a longer health education curriculum of 24 sessions over 8 weeks) can lead to changes in risk perception and information-seeking behaviors towards air pollution in the intervention group. However, this study included a longer-term educational activity, and their survey lacked any specific questions for assessment of changes in relevant environmentally friendly behavior. Another study, conducted between 2000 and 2001 at Dalhousie University, Canada, delved into the behavioral effects of environmental education in college students. Its focus was on gauging the impact of an environmental studies course, which included every other week seminars and twice-weekly lectures during two semesters. To measure the effect, the researchers administered a pre-existing questionnaire developed by Kempton et al. (1995), both before and after the course duration. Notably, the findings showed a noticeable improvement in environmentally responsible behavior after the conclusion of the course. Nevertheless, this was a longer educational program, with no long-term follow up beyond the conclusion of the course and lacked any control arm.”

5- *COMMENT: The authors state that the teaching session was based on available literature but what available literature? Are they articles on air pollution, teaching methods, or both? There is also a lot of pre-existing lesson plans for environmental education. How do these sessions compare to those?*

RESPONSE: We apologize if this was not clarified. The teaching sessions’ contents were designed to provide evidence-based information about the air pollution and its health effects (or vaccination and its benefits for the control group). The focus of the study was to deliver such evidence-based presentations to adolescents, and not on how it was delivered to them, with the rationale that the adolescents will better respond to the environmental education.

We have revised the manuscript to better clarify this point.

Methods section, Page 13-14

" The teaching sessions were one hour in duration, script-based, and associated with a PowerPoint slide presentation (https://arjomandilab.ucsf.edu/questionnaires-powerpoints). The intervention (air pollution) teaching session was developed by our research group, which comprised researchers and educators including an educator from the San Jose KIPP school, and based on the available scientific literature on air pollution and its health effects from the United States Environmental Protection Agency and World Health Organization.[19, 20] The session involved an interactive session that included undergoing a breathing test (spirometry) using a handheld spirometer (EasyOne spirometer; ndd Medical, Inc., Zurich, Switzerland). The Control (vaccination) session also involved an evidence-supported script-based session developed by our research group based on the information on vaccination and its benefits available from the United States Centers for Disease Control website and The Public Broadcasting Company.”

*6- COMMENT: Does the study population live in an area of high air pollution (I believe it does since they are in San Jose)? It would be useful to mention that as well as some summary statistics of air pollution levels and asthma rates or other childhood illnesses due to air pollution within the study area.*

RESPONSE: We have provided a comment about the level of air pollution in San Jose area as requested.

Methods section, Page 11

“… Air pollution levels are consistently higher in the South San Francisco Bay and in the San Jose Metropolitan area compared to the neighboring parts. For example, the highest average pollutant concentration for ozone (over an eight-hour period) and particulate matter of 10 micron or less (over a 24-hour period) were 81 ppb and 77 mcg/m^3^, respectively, in San Jose in 2019. By comparison, those levels were 73 ppb and 42 mcg/m3 in San Francisco.”

*7- COMMENT: Is one month after the teaching session sufficient to show permanent behavior changes? Where did this time frame come from?*

RESPONSE: The original design of the study was to have longer term follow up of the participants including follow-up at 3, 6, and 12 months with hoped that further follow ups could also be performed. To allow for holidays and scholastic schedule of the students, the questionnaire may have been administered within two months of the planned 3-, 6-, and 12-month timelines. In the case of the current participants, the questionnaire was administered at about one month after the teaching sessions (36 to 47 days after the teaching session).

We have revised the manuscript to clarify the questionnaire timeline better.

Methods section, Page 10

“…. To quantify the effect of the teaching session, we developed a survey questionnaire with two topic domains to measure the understanding and future behavioral choices of the participating adolescents towards air pollution. To determine the longitudinal effectiveness in promoting environmentally friendly behavior, we planned to administer the questionnaire immediately before, immediately after and then at intervals of three, six, and twelve months. To allow for scholastic holidays, the questionnaire timeline was allowed to be administered within two months before or after the planned timelines (e.g., one to five months for three-month time point) …”

Results section, Page 19

“The study enrollment began on February 1, 2015 and students from three classrooms at San Jose KIPP school constituted the first wave of enrollment. Although the study remained open to enrollment until December 20, 2018, no further students were enrolled in the study due to funding limitations. The study only enrolled students from a single school and during one school year and the enrolled students completed the same survey only immediately before, immediately after, and at about one month (36 to 47 days after the teaching session).”

*8- COMMENT: If this is mainly a Hispanic population were the questionnaires also administrative in Spanish?*

RESPONSE: The consent and ascent forms were provided in English and Spanish to account for possibility that the students’ parents or guardians were not able to read and understand English. However, the teaching sessions and the questionnaire administered to the students were only in English. The KIPP students were all proficient in English language at the level of middle school. Ability to read/understand/write in English was a required criteria for enrollment in the study.

We have clarified this in the manuscript.

Methods section, Page 11-12

“Students who provided signed consent and assent forms were included in the study if they met the enrollment criteria. The consent and ascent forms were provided in English and Spanish language to allow for language preference of the students’ parents and/or guardians in this school with majority Hispanic population. However, the teaching sessions and the questionnaire administered to the students were only in English, as the KIPP students were considered to all be proficient in reading and writing English language at the level of middle school. The study enrollment ended on December 20, 2018.”

*9- COMMENT: Is a one-hour teaching session really going to that effective? Would it not be better to design a longer course?*

RESPONSE: This was exactly what the study was designed for! It is the main research question. To test to see how effective a one-time teaching session could be in the long term. Our findings suggest that even a short, limited teaching session could have effects at one month and possibly beyond. Unfortunately, the longer-term time points for administration of the questionnaire were not achieved.

**REVIEWER #3**

*1- COMMENT: There are no line numbers so comments provided below with ref to page numbers.*

RESPONSE: Line numbers are added.

*2- COMMENT: Abstract Background - revise statement re “daily living choices” – be more specific since this current sentence is not helpful, and also does not help improve understanding of claims in Abstract “Discussion” sentence re “env friendly behavior choices”.*

RESPONSE: We have revised the wording and provided a hopefully more helpful description of the rationale.

Abstract section, Page 6

“**Background-**Despite the wealth of scientific information on the health effects of air pollution, the adult public’s lifestyle continues to be largely detrimental towards the environment.”

*3- COMMENT: Abstract Objective – add for clarity “could shift REPORTED behavioral choices”.*

RESPONSE: Revised accordingly.

Abstract section, Page 6

“… could shift reported behavioral choices of adolescents towards environmentally friendlier options.”

*4- COMMENT: Abstract Methods – for added clarity add “ACTIVE control group”.*

RESPONSE: Revised accordingly.

Abstract section, Page 6

“… on either the effects of air pollution on lung health (intervention group) or the role of vaccination in public health (active control group).”

*5- COMMENT: Abstract Methods – An important potential limitation is the metric used for evaluation since the survey was developed for this study but there is little info on this in the main text, for the abstract suggest making this clearer that the survey was new/designed for this study…”.*

RESPONSE: Revised accordingly.

Abstract section, Page 6

“… newly designed for this study to evaluate …”

*6- COMMENT: Abstract Results – perhaps another measure to report re income since range is so wide for median?*

RESPONSE: The income level was obtained using categories including <25K, 25K-50K, etc. and thus reporting IQR is not possible; however, we have provided an additional measure.

Abstract section, Page 6

“…median annual family income=$25K-$50K with 70.1% <$50K…”

*7- COMMENT: Introduction– clarify what is meant, or give a few examples parenthetically, re “personal choices in their daily lives” – same issue with second paragraph – and which populations are you targeting/referencing? USA specifically or high-income countries, pls clarify too.*

RESPONSE: Revised accordingly.

Introduction section, Page 8

“… personal choices in their daily lives (for example, use of public versus private transportation or use of locally produced versus imported food) …”

Introduction section, Page 8

“… (for example, economic prosperity and reducing healthcare costs are considered of much higher priority as opposed to protecting environment and dealing with climate change). Interestingly, at least in the developed countries, such attitudes seem not to be necessarily governed by income levels...”

*8- COMMENT: Introduction– Sent beginning “One potential” – this is too broad of an assertion, suggest revising to “One potential FACTOR ASSOCIATED WITH the disconnect…”.*

RESPONSE: Revised accordingly.

Introduction section, Page 8

“One potential factor associated with the disconnect between the state …”

*9- COMMENT: Methods- If this was a “pilot” study, make this clearer earlier on not just in the discussion (helps explain the relatively small n).*

RESPONSE: This was a pilot study, which we hoped and tried to expand to make a full study, but was unable to do so due to funding limitation. We have revised the manuscript as recommended to clarify this point by specifying its pilot nature in the title and other parts of the manuscript.

*10- COMMENT: Methods- More info on HOW the one-hour teaching session was developed is needed – details can be provided in the SI (there is currently a link to slides, but this should be provided in the SI).*

RESPONSE: We have revised the manuscript to include additional information about how the teaching session and the questionnaire were developed, as was also recommended by Reviewer 2 (Comments #2 and #5) and the teaching session content, and questionnaire along with its scoring metrics, is submitted as part of the protocol which is being deposited on Protocols.io server, which is awaiting DOI assignment.

*11- COMMENT: Methods- Given the centrality of the survey instrument to evaluation and outcomes it should be provided as part of the SI.*

RESPONSE: We have now uploaded the study protocol including the questionnaire and methods for scoring it in Protocols.io, and is awaiting the assignment of a DOI. Additionally, the protocol has been submitted as a supplement.

*12- COMMENT: Methods- How were the three classrooms selected? Provide more info/details – and to confirm: three classrooms of students from one school, yes? Also re the timeframe was this study rolled out to each classroom simultaneously or otherwise. Explanation is needed generally and especially in light of the nearly four-year time period of the study (?).*

RESPONSE: We have provided additional information about the selection of the classrooms/school and timeframe. All three 8^th^ grade classrooms at the San Jose KIPP school were chosen simply due to convenience as we had familiarity with the teachers at that school- a previous study coordinator from our lab, joined Teach for America and then joined the San Jose KIPP school as a teacher. Thus, San Jose KIPP was chosen as a matter of convenience with plans to engage other schools and teachers from other San Francisco Bay Area School Districts (including San Jose, San Francisco, Oakland, etc. at a later time).

Methods section, Page 11

“…As an initial attempt, teachers from the San Jose, California metropolitan area KIPP school (Knowledge Is Power Program), a national network of free open-enrollment college preparatory public schools dedicated towards students in underserved communities, were invited to participate in the study by collaborating with study recruitment and educational activities. Air pollution levels are consistently higher in the South San Francisco Bay and in the San Jose Metropolitan area compared to the neighboring parts. For example, the highest average pollutant concentration for ozone (over an eight-hour period) and particulate matter of 10 micron or less (over a 24-hour period) were 81 ppb and 77 mcg/m3, respectively, in San Jose in 2019. By comparison, those levels were 73 ppb and 42 mcg/m3 in San Francisco.

Beginning February 1, 2015, students from all three 8th grade classrooms at the San Jose KIPP school were invited by their teachers to participate in the study. The interested students were provided with a letter of invitation along with assent and consent forms that they took home to review with their parents. Students who provided signed consent and assent forms were included in the study if they met the enrollment criteria. The consent and ascent forms were provided in English and Spanish language to allow for language preference of the students’ parents and/or guardians in this school with majority Hispanic population. However, the teaching sessions and the questionnaire administered to the students were only in English, as the KIPP students were considered to all be proficient in reading and writing English language at the level of middle school. The study enrollment ended on December 20, 2018.”

Results section, Page 19

“The study enrollment began on February 1, 2015 and students from three classrooms at San Jose KIPP school constituted the first wave of enrollment. Although the study remained open to enrollment until December 20, 2018, no further students were enrolled in the study due to funding limitations. The study only enrolled students from a single school and during one school year and the enrolled students completed the same survey only immediately before, immediately after, and at about one month (36 to 47 days after the teaching session).”

*13- COMMENT: Methods- Provide more info/details re “simple randomization” What was the breakdown re treatment/control by classroom following randomization?*

RESPONSE: The students were randomized such that about equal numbers of students in each classroom were assigned to intervention and control groups. We have provided clarification in the revised manuscript.

Methods section, Page 12-13

“The participating students were assigned a study ID number through sorting their last names (and first names if needed) alphabetically and then assigning each student a study ID number. They were then partitioned by simple randomization of their subject ID number within their classrooms using STATA software to either the intervention (air pollution teaching) or control (vaccine teaching) group such that about equal numbers of students in each classroom were assigned to intervention and control groups.”

*14- COMMENT: Methods- So teachers facilitated recruitment and enrollment? If so state clearly, if not explain.*

RESPONSE: The reviewer is correct in their understanding that the teachers facilitated recruitment and enrollment by first informing the students about the study and then distributing the consent and ascent forms to the interested students, and later collecting the forms.

Methods section, Page 11

“Beginning February 1, 2015, students from all three 8th grade classrooms at the San Jose KIPP school were invited by their teachers to participate in the study. The teachers then distributed a letter of invitation along with assent and consent forms to the interested students to take home and review with their parents/guardians, and later collected and provided the forms to the study investigators.”

*15- COMMENT: Methods- explain “spirometry maneuver”.*

RESPONSE: Revised accordingly.

Methods section, Page 14

“… that included undergoing a breathing test (spirometry) using a handheld spirometer…”

*16- COMMENT: Methods- were any educators, or education researchers, involved or consulted in the creation of the teaching session – explain either way (if not, add to limitation sections).*

RESPONSE: Yes. As mentioned above, one of the teachers involved, a co-author on the manuscript, was an educator at KIPP school and played an active role in designing the teaching session and the questionnaire.

Methods section, Page 13

“… The intervention (air pollution) teaching session was developed by our research group, which included researchers and educators including an educator from the San Jose KIPP school, and based on the available scientific literature on air pollution and its health effects.”

Methods section, Page 14

“The questions on the questionnaire were formulated collaboratively by our research team that included researchers and educators (including an educator from the San Jose KIPP school).”

*17- COMMENT: Methods- was there any testing/evaluation of the survey-questionnaire (psychometrics)? Explain either way and if not add to limitations.*

*Methods- assuming this evaluation instrument has not been used before, pls add some text to contextualize the use of such a tool and resulting scores/scales for this type of evaluation.*

RESPONSE: We have provided details on assessment of the reliability and validity of the questionnaire in the revised manuscript, as mentioned above in response to comment #2 of the Reviewer 1- see below.

Methods section, Questionnaire- Page 16

“…The structural validity (reliability) of the questionnaire was evaluated by computing the correlation coefficient of the data collected before and immediately after the teaching sessions from all participants. To account for any potential effects from the intervention administered, the correlation coefficient of the data was also computed within the control group. The content validity was evaluated by factor analysis with “varimax” rotation using data collected from all participants before the teaching sessions. Barlett’s test of sphericity was performed to test whether data was appropriate for factor analysis. The number of factors to extract from factor analysis was determined using Scree plot and parallel analysis. Factor loadings and percentage of variance explained by the factors were reported.”

Results section, Questionnaire- Page 23-24

Questionnaire Performance

“…The test-retest reliability of the questionnaire computed using data collected from all participants before and immediately after the teaching sessions was 0.77. The test-retest reliability was 0.75 when it was computed using data from the control group only. Barlett’s test of sphericity showed a significant P value <0.001and thus granted appropriateness of factor analysis. Scree plot and parallel analysis of data suggested factor analysis with two factors. This analysis showed that using two factors separated the survey questions in accordance with the understanding and future behavior domains but explained only 19.4% of the variance (Supplemental Table S1). We subsequently performed factor analyses for three, four, and five factors. With five factors, the percentage of variance explained by the factors improved to be 43.1% with survey questions quantifying behaviors loaded mostly on Factors 1, 3 and 4; while questions quantifying understanding loaded mainly on Factor 2 (Supplemental Table S2).”

*18- COMMENT: Methods- re regression, where any other variables other than age, sex, ethnicity available? – eg, income data is mentioned previously and reported in Table 1, why was this not also used? (later mentioned in the discussion but “data not shown”).*

*Methods- Related, was there a pre-specified statistical analysis plan? If yes, what covariates were pre-specified? If not, add to limitations.*

RESPONSE: We have provided further details about the regression analyses and justification of the variables included in the revised manuscript, as described above in response to comment #9 of the Reviewer 1- see below. All data has now been included and discussed in the manuscript and is uploaded on Dryad.

Methods section- Page 17-18

“… Regression analyses using generalized estimating equations (GEE) were also performed to examine the changes in outcomes with a repeated measure design and adjustment for covariates including age, sex, race, ethnicity, income level, and assigned classrooms as appropriate. Given that only few students provided race classification (22 out of 77) and the skewed distribution of family income levels of participants, only age, sex, and ethnicity were included in the final regression modeling. Furthermore, linear regression modeling with adjustment for covariates was performed for comparison of changes between the intervention and the control group at different time points.”

*19- COMMENT: Methods- No mention of power calculations – pls add or add to limitations or both.*

RESPONSE: We have provided details of sample size and power calculation in the revised manuscript, as described above in response to comment #9 of the Reviewer 1- see below.

Methods section, Sample Size and Power Calculations- Page 16

**Sample size and power calculation**

“For sample size and power calculation, we made the following assumptions: (1) minimally important difference in questionnaire score of 10% change due to the intervention, (2) standard deviation of change in score of 40%, and (3) a drop-out rate of about 20% due to factoring including movement of subjects out of the area. A sample size of 504 subjects (252 subjects in each group) will provide a power of 80% to detect a change in questionnaire score by t-test with a two-sided type I error of 0.05. Considering the drop-out rate, we proposed to recruit a total of 600 subjects (300 in each group).”

*20- COMMENT: Results- given the potential limitations associated with the evaluation metric and range used, it is difficult to determine whether the small statistically sign difference is in fact clinically significant or not….*

*Based on the available information and data, I don’t think there is sufficient evidence to claim that these observed differences are a “significant and important increase” …more info, as requested above, about the evaluation instruments might help in this regard…*

RESPONSE: Given the novel nature of the questionnaire, we used the data collected at baseline during this study to determine a range of possible MIDs, as detailed in the manuscript. While we settled on a value of 1 as the chosen MID, it is worth noting that the statistically significant improvement in the scores that we observed following the intervention were larger than most estimates of possible MIDs calculated. We agree that one should be cautious in interpreting the findings of the study given the samples size and other limitations; however, the statistical analysis of the data is robust regardless of the approach and provides confidence in the findings. As suggested by the reviewer, in the revised manuscript, we have pursued a more cautious tone in our interpretation of the findings.

Abstract, Page 6

“However, the intervention (air pollution) teaching session resulted in a statistically significant increase in behavior domain score from baseline to immediately post-teaching, which continued to be present at one-month follow-up (mean±SD of score change immediately after=1.7±3.3; score change 1-month after=2.5±3.2; P<0.001; minimally important difference=1.0).”

Results section, Page 25

“… However, the intervention (air pollution) teaching session did result in a statistically significant increase in behavior domain score from baseline to immediately post-teaching, which was greater than the presumed MID for the questionnaire. Of note, this improvement continued to be present at 1-month follow up”

*21- COMMENT: Results- Again, why no adjustment for reported family income?*

RESPONSE: We have provided the rationale in the manuscript, as detailed above in response to the Reviewer 3 Comment #18.

*22- COMMENT: Results- Suggest removing word “Remarkably” since it’s likely not appropriate given the design limitations and sample size…*

RESPONSE: Removed. Now it says “interestingly”.

Results section, Page 28

“Interestingly, at 1-month follow-up survey, there was a significant increase from baseline in the behavior domain score of the control group…”

*23- COMMENT: Results- Also the observed difference in the C group, if anything, adds contextual evidence that the observed difference in the T group discussed is likely not clinically significant… (re discussion: given the population and nature of the intervention, the assumption that this might have been due to spillover seems unlikely – but given the way the intervention was delivered it’s of course possible)…*

RESPONSE: The reviewer’s point may be valid if we look at the scores from the 1-month follow up survey in isolation; however, looking at the larger data including the baseline data, the provided reliability and validity analysis, and calculated estimates of MID, the reviewer’s point is less likely to be correct.

For example, while the reviewer’s comment could provide a possible explanation in case of the similarity of the 1-month follow up scores, it does not provide a reasonable explanation for the change in the scores observed immediately post-teaching. The difference in scores that we observed immediately following the teaching session were statistically significantly different between the intervention and the control groups (1.7 versus 0.6), and the change in score in the intervention group was larger than most estimates of MIDs calculated. This was not the case for the control group. Thus, it is more appropriate to conclude that immediately after teachings session, environmental education (versus education about vaccine) likely improved the responses towards environmentally friendly outcomes. In addition, at 1 month follow up, the changes in scores for both groups were larger than most estimates of possible MIDs calculated. Given the nature of randomization process, we agree with the reviewer that the spillover effect is a possible explanation, but disagree with their alternate explanation that the score change was not clinically significant.

*24- COMMENT: Discussion- There are many methodological limitations that need to be more clearly discussed. The authors should create a dedicated Limitations section (as of now, limited discussion of limitations is somewhat scattered) or paragraph/s in the discussion section to address some the following and other points raised above: …*

RESPONSE: We have generated a “Limitations” section, as requested, and included additional limitations commented on by the reviewers.

*25- COMMENT: Discussion- Pls provide some info about the 25 students who declined ascent/consent and if/how they appeared to differ from those participating – if info not available pls state…*

RESPONSE: The characteristics of the students who participated in the study are representative of the KIPP student population in general. Those 25 students and their parents who declined participation, however, did not provide ascent and consent, and because of that, no data was collected from them and no comment could be made about their characteristics. Such data collection or reporting would have been inappropriate and a violation of IRB guidelines. We disagree that any comments about the lack of information about those who did not provide ascent/consent should be made.

Limitation section- Page 33

“… Furthermore, the non-response rate was relatively high (near 25%). Moreover, the longitudinal follow-up was confined to one long-term assessment timepoint after the initial visit assessment. Nevertheless, the uniform demographics and socioeconomic status of the student population, coupled with the minimum loss to follow-up likely counter the concerns for non-response and survivorship biases.”

*26- COMMENT: Discussion- Why conducted over almost four years if only three classrooms and short follow-up?*

RESPONSE: The goal of the study was to recruit over 600 students as detailed in the sample size calculation and listed on the ClinicalTrials.gov. The goal of the study was also to follow up with the enrolled students on several occasions for one year and possibly beyond. However, we were not able to obtain adequate funding to continue with the study. Several grant applications were made with the hope that funding could be secured and the study could be expanded, but unfortunately those attempts were not successful. At the end, we were left with having to abandon the study.

We have Provided further details in the Methods and Results section to clarify this.

Results section, Page 19

**Participants’ Characteristics**

“The study enrollment began on February 1, 2015, and students from three classrooms at San Jose KIPP school constituted the first wave of enrollment. Although the study remained open to enrollment until December 20, 2018, no further students were enrolled in the study due to funding limitations. The study exclusively enrolled students from a single school within the span of one academic year. These enrolled students completed the same survey solely immediately before, immediately after, and at about one month (36 to 47 days after the teaching session).”

*27- COMMENT: Discussion- With such a relatively small n it may have made sense to use block randomization to better balance covariates (eg, income variable) – this is kind of mentioned re current discussion of cluster design (note that this would actually not be appropriate if only three classrooms) - should be discussed with other limitations.*

RESPONSE: We apologize if the description of the study randomization was not explained well. The study did in fact used a block randomization. The participating students from each class were listed alphabetically and then assigned a subject ID. The subject IDs were then used for random sampling using STATA such that equal number of students in each class were assigned to intervention or control group. We have provided clarification in the Methods section.

Methods section, Page 12-13

“The participating students were assigned a study ID number through sorting their last names (and first names if needed) alphabetically and then assigning each student a study ID number. They were then partitioned by simple randomization of their subject ID number within their classrooms using STATA software to either the intervention (air pollution teaching) or control (vaccine teaching) group such that about equal numbers of students in each classroom were assigned to intervention and control groups.”

Discussion section, Page 33

“… A cluster-based approach with all students within each class receiving the same intervention could have possibly helped to avoid a spillover effect if larger number of classrooms were included..”

*28- COMMENT: Discussion- Some discussion re difference between T and C groups for those <25k income should be added.*

RESPONSE: Although there was a statistically significant difference in the number of lower family income (income <$25K) randomized into the intervention vs. the control group (41% in the intervention group vs. 22% in the control group), adjustment for level of family income (when evaluated by the 5 income level categories or when dichotomized into < and >$25K) did not show any significant difference in outcomes between intervention and control groups. However, this study was not powered to look at the effect of income level (even at 600 participants originally planned sample size), and as such, no conclusion could be made about the lack of association from the current data.

We have provided further clarification about this limitation of our study and its inability to draw any conclusion on the effect of income level on outcomes.

Limitation section, Page 35

“Finally, there was a limited span of the income level among the population studied with about two thirds of the participants reporting annual income levels below $50,000 (at or near poverty level in the US). This limitation may adversely affect the generalizability of the study findings, as the income level could potentially influence people’s behavior towards environment. However, our study was not powered to examine the contribution of socioeconomic factors in efficacy of the intervention administered.”

*29- COMMENT: Discussion- One of the primary limitations – not unique to this study – is the use of a custom-designed survey and also study-specific scores/metrics to evaluate the outcomes of interest – both present substantial opportunities for bias – associated issues and limitations (re responses to questions above) should be discussed.*

RESPONSE: We have provided additional discussion about this possible limitation under Limitations in the Discussion section.

Limitation section, Page 34

“… Custom-designed surveys could be prone to various biases in evaluation of the outcomes of interest, including but not limited to sampling, non-response, and survivorship biases among others. Our pilot study is not exempt from some of these potential biases. For instance, the study had a small sample size, and it was conducted within a single school with a relatively narrow demographic representation. Furthermore, the non-response rate was relatively high (near 25%). Moreover, the longitudinal follow-up was confined to one long-term assessment timepoint after the initial visit assessment. Nevertheless, the uniform demographics and socioeconomic status of the student population, coupled with the minimum loss to follow-up likely counter the concerns for non-response and survivorship biases. Furthermore, biases can potentially arise from the specific metrics used to score such surveys, such as question order, answer arrangement, and conformity biases. However, the orders of the questions and answers on the survey had been in fact mixed, mainly to prevent any possibility of the participants copying answers from each other, but it also served to alleviate concerns associated with any biases stemming from question or answer order. Lastly, open-ended questions were incorporated, along with their specific scoring methodology, to reduce the effect of any conformity and social desirability bias.…”

*30- COMMENT: Discussion- As it stands, not enough evidence/info to support claim that a main strength of the study was “development of teaching materials and questionnaire” – indeed this could be construed as a primary weakness – more information is needed so the reader can better understand/decide.*

RESPONSE: In the revised submission, we have included additional supporting information including the validity and reliability analysis along with the complete study protocol (that includes the questionnaire and its scoring metrics), the complete dataset, and further methods, results, and discussions based on the reviewers’ inputs. We also have removed the reference to the teaching and questionnaire as a strength of the study.
